# 

**Published:** 1909-02

**Authors:** 


Sixty-fourth Year.

VOL. LXIV. FEBRUARY, 1909. No. 7

### BUFFALO MEDICAL JOURNAL

LIBRARY
Indexed
S. G. O.

Established 1845 by AUSTIN FLINT M. D.

SURGEON GENERAL'S OFFICE
FEB 1 - 1909

EDITOR:
WILLIAM WARREN POTTER, M. D.
ASSISTANT EDITORS:
WILLIAM C. KRAUSS, M. D. NELSON W. WILSON, M. D.
ASSOCIATE EDITORS:
JAMES WRIGHT PUTNAM, M. D. ERNEST WENDE, M. D.
JOHN PARMENTER, M. D. JOHN A. MILLER, PH. D.
MAUD J. FRYE, M. D.

